# Publisher Correction: FIPRESCI: droplet microfluidics based combinatorial indexing for massive-scale 5′-end single-cell RNA sequencing

**DOI:** 10.1186/s13059-023-02944-7

**Published:** 2023-04-21

**Authors:** Yun Li, Zheng Huang, Zhaojun Zhang, Qifei Wang, Fengxian Li, Shufang Wang, Xin Ji, Shaokun Shu, Xiangdong Fang, Lan Jiang

**Affiliations:** 1 China National Center for Bioinformation, Beijing, 100101 China; 2 CAS Key Laboratory of Genome Sciences and Information, Beijing Institute of Genomics, Chinese Academy of Sciences, Beijing, 100101 China; 3 University of Chinese Academy of Sciences, Beijing, 100049 China; 4 Sino-Danish College, University of Chinese Academy of Sciences, Beijing, 100049 China; 5 The Blood Transfusion Department, First Medical Center of Chinese, PLA General Hospital, Beijing, 100853 China; 6 Key Laboratory of Carcinogenesis and Translational Research (Ministry of Education/Beijing), Gastrointestinal Cancer Center, Peking University Cancer Hospital & Institute, No. 52 Fucheng Road, Beijing, 100142 China; 7 Peking University International Cancer Institute & Peking University Cancer Hospital & Institute, Beijing, 100191 China; 8 Institute for Stem Cell and Regeneration, Chinese Academy of Sciences, Beijing, 100101 China; 9 Beijing Key Laboratory of Genome and Precision Medicine Technologies, Beijing, 100101 China; 10 College of Future Technology College, University of Chinese Academy of Sciences, Beijing, 100049 China

**Publisher Correction: Genome Biol 24, 70 (2023)**

**https://doi.org/10.1186/s13059-023-02893-1**

Following publication of the original article [1], the authors identified a typesetting error in Fig. 1. While processing the figure, some layers were lost.

**Fig. 1:**
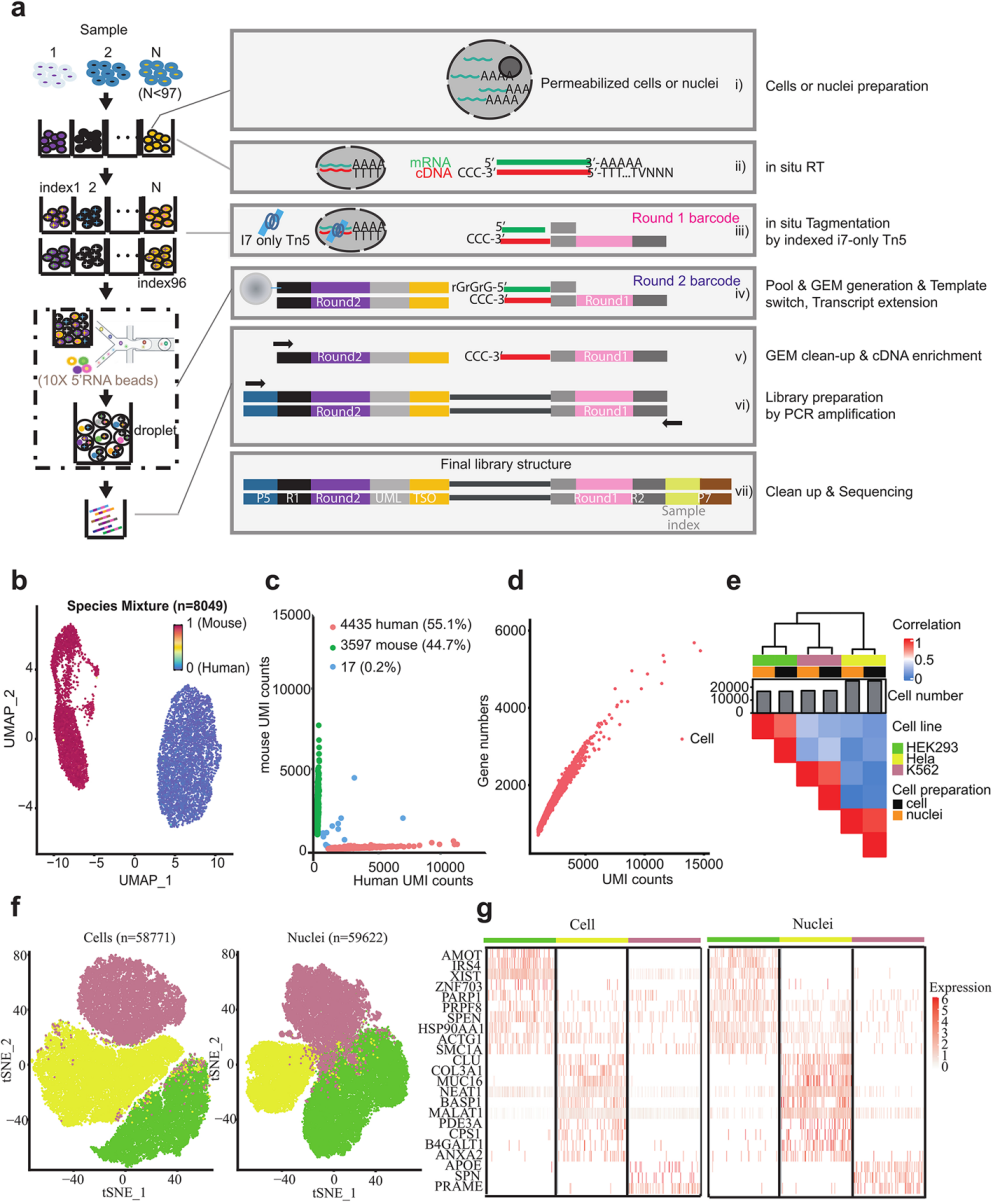
Overview and validation of FIPRESCI. **a** The FIPRESCI schematic workflow and detailed method design. Permeabilized cells or nuclei are reverse transcribed, then nuclei or cells are randomly distributed into wells containing indexed Tn5 transposome to label the cellular origin of RNA/cDNA hybrid heteroduplexes within cells. The cells or nuclei containing preindexed cDNA are pooled, randomly mixed, and encapsulated using a commercial microfluidic platform and amplified for preparation of the sequencing library. **b** Species-mixing experiment with a library prepared from the 1:1 mix of human (Jurkat) and mouse (NIH-3T3) permeabilized cells. Human uniquely barcoded cells (UBCs) are blue, mouse UBCs are red in UMAP. *n* = 8049 cells. **c** The number of unique fragments aligning to the human or mouse genome. Human UBCs are red, mouse UBCs are green, and mixed-species UBCs are blue. The estimated barcode collision rate is 0.2%, whereas species purity is > 99%. **d** The number of UMI counts plotted against detected genes from species-mixing experiments. **e** Heatmap showing pairwise correlations and hierarchical clustering for the gene expression profiles across cell lines, cell preparation methods using FIPRESCI. **f** Dimensionality reduction (UMAP) and unsupervised clustering for single-cell (*n* = 58,771) and single-nucleus (*n* = 59,622) FIPRESCI of the three cell lines. HEK293 is red, Hela is green, and K562 is blue. **g** Heatmap showing differentially expressed genes and gene expression levels of single-cell and single-nucleus FIPRESCI for three cell lines. Each column represents a single cell

Further to this, the authors would like to add the following statement to the Acknowledgement section: This publication is part of the Human Cell Atlas—www.humancellatlas.org/publications.

The correct Fig. 1 is given in this correction and the original article [1] has been corrected.
