# Supplementary material for: FIPRESCI: droplet microfluidics based combinatorial indexing for massive-scale 5′-end single-cell RNA sequencing
Source: Genome Biol. 2023 Apr 6;24:70. doi: 10.1186/s13059-023-02893-1 (PMC10078054; doi:10.1186/s13059-023-02893-1)
Supplement: Supplementary file 1 — Additional file 1: Figure S1-S13. [file 13059_2023_2893_MOESM1_ESM.pdf]

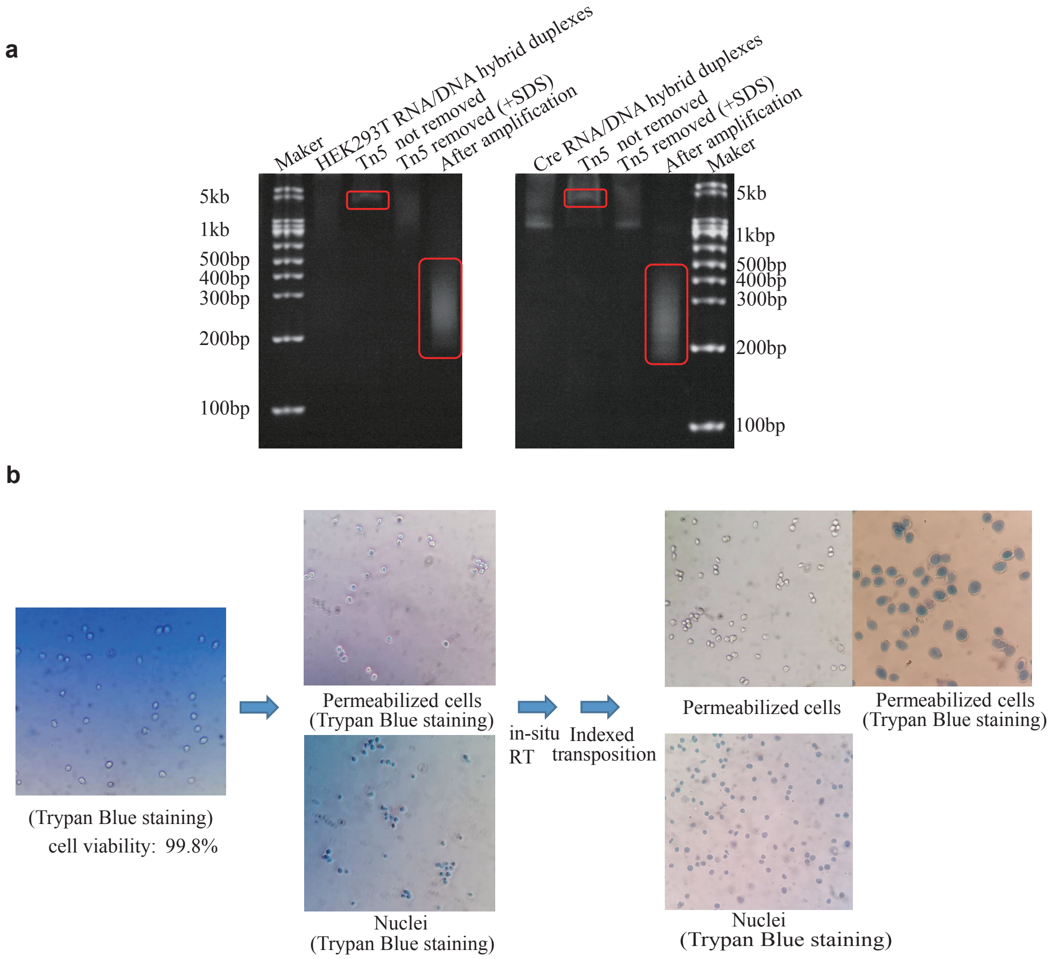

Fig. S1 | The Tn5 transposase maintains the contiguity of target RNA/DNA hybrids after transposition.

a. PAGE analysis of the contiguity of transposase-targeted RNA/DNA hybrids. The Tn5 transposome was used to target RNA/DNA hybrids (the reverse transcription product of HEK293T mRNA (left) and Cre RNA (right) respectively). After transposition, RNA/DNA hybrids were either treated with SDS to remove the transposase enzyme or not treated as a control. Lane 1 (left) and lane 5 (right) shows a 100-bp plus reference ladder. The Tn5 transposase enzyme stays bound to its substrate RNA/DNA hybrids after transposition, and the protein-DNA complex only dissociates after the addition of a protein-denaturing agent (SDS). Lane 5 (left) and lane 4 (right) shows the amplification product of fragmented RNA/DNA hybrids.

b. Figures show the intact structure of nuclei and permeabilized cells (HEK293T) after reverse transcription reaction and indexed transposition under the microscope.

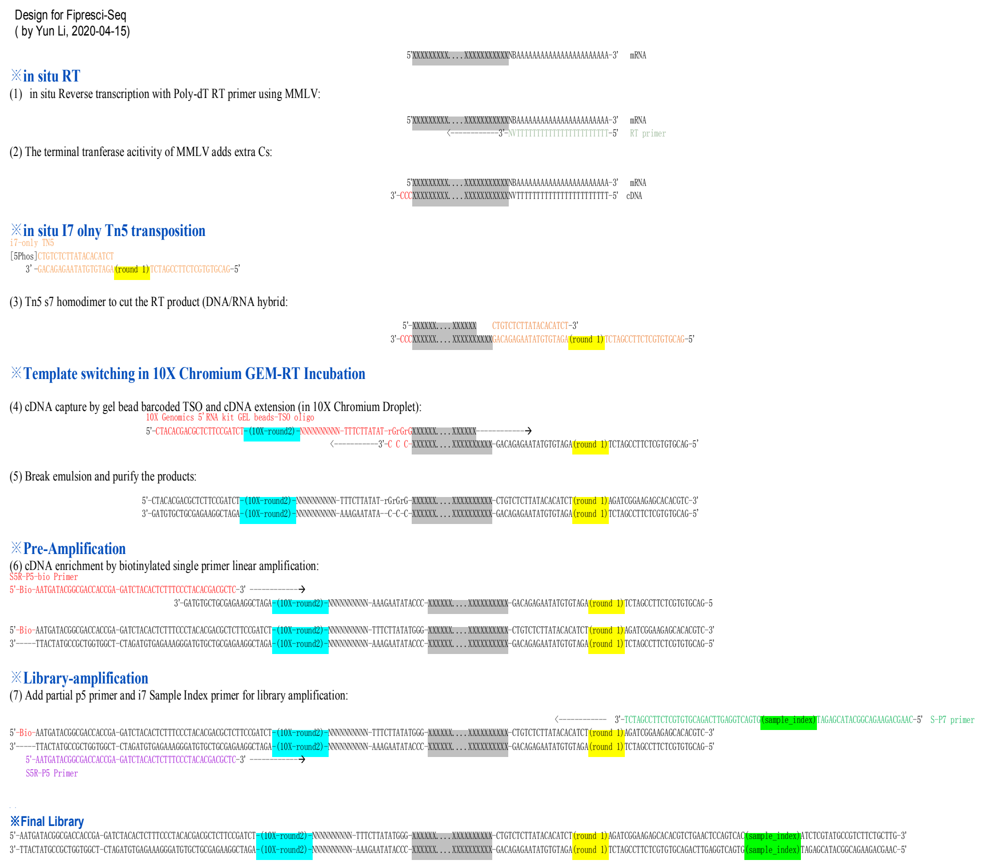

Fig. S2 | Detailed assay design of FIPRESCI.

Schematic outline of FIPRESCI including detailed oligonucleotide sequences. The reverse transcription is performed inside permeabilized cells or nuclei. Then permeabilized cells or nuclei are distributed on a 96-well or 384-well plate containing indexed Tn5 transposase introducing well-specific round1 barcodes into the whole transcriptome. Pre-indexed cells or nuclei are pooled and encapsulated into emulsion droplets using a standard microfluidic droplet generator (10x Genomics Chromium). The round2 barcodes are introduced by template-switching and transcript extension with barcode Template Switching Oligo (TSO) within Gel-beads ((10x Genomics Chromium, 10x Genomics Single Cell 5' RNA Reagent Kits). The droplet emulsion is then broken, and cDNA labeled with round1 and round2 are enriched by PCR amplification and prepared for sequencing. Finally, the library is PCR amplificated, with the option to introduce an additional sample index.

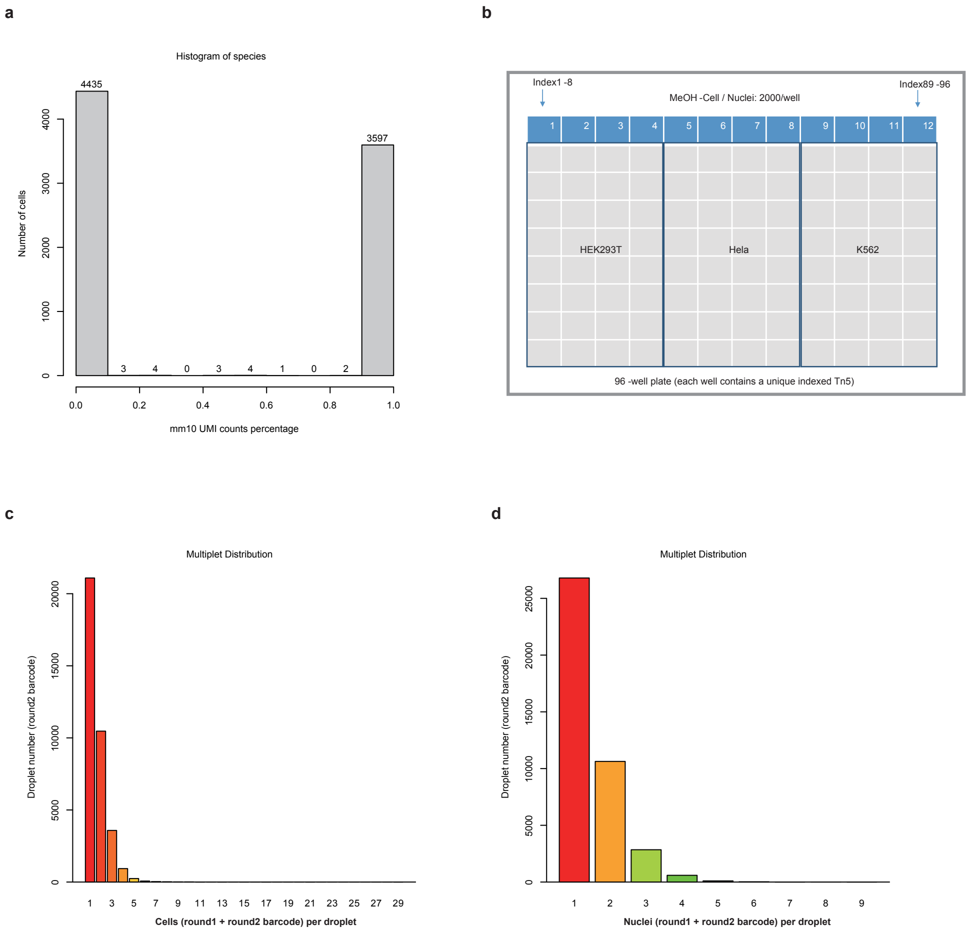

Fig. S3 | FIPRESCI enables massive-scale single-cell experiments.

a. Histogram showing the frequency and distribution of reads aligning to the human or mouse genome in species mixing experiments.

b. Design and indexed TN5 usage for three cell lines validation experiments.

c. Distribution of the number of cells per droplet when loading 100k cell.

d. Distribution of the number of nuclei per droplet when loading 100k nuclei.

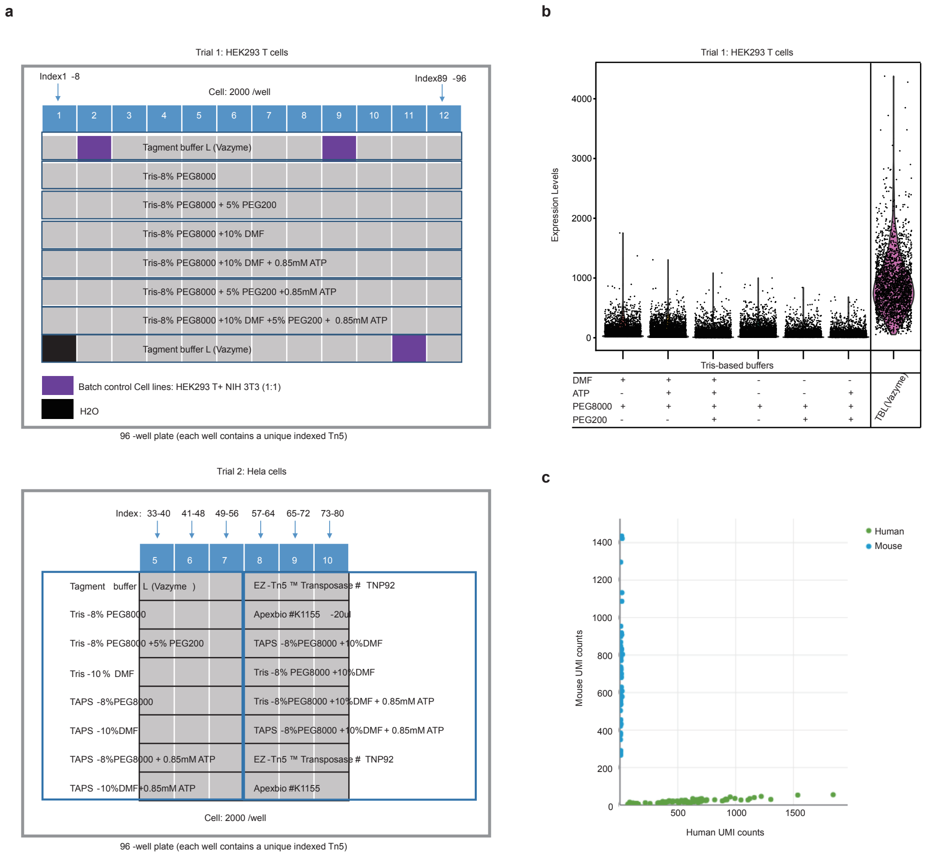

Fig. S4 | Design and performance of different tagmentation buffers in FIPRESCI.

a. Design and indexed TN5 usage for different tagmentation buffers for FIPRESCI. Two independent trials have been done. The detailed components of these tagmentation buffers are provided in Supplementary Table 3.

b. Violin plot showing sensitivity in FIPRESCI generated with the Trial 1 TN5 tagmentation buffers. Each dot represents a single cell. Y-axis is the number of genes detected.

c. The number of unique fragments aligning to the human or mouse genome from batch control data in Trial 1 (Supplementary Fig5.a (upper) the purple wells).

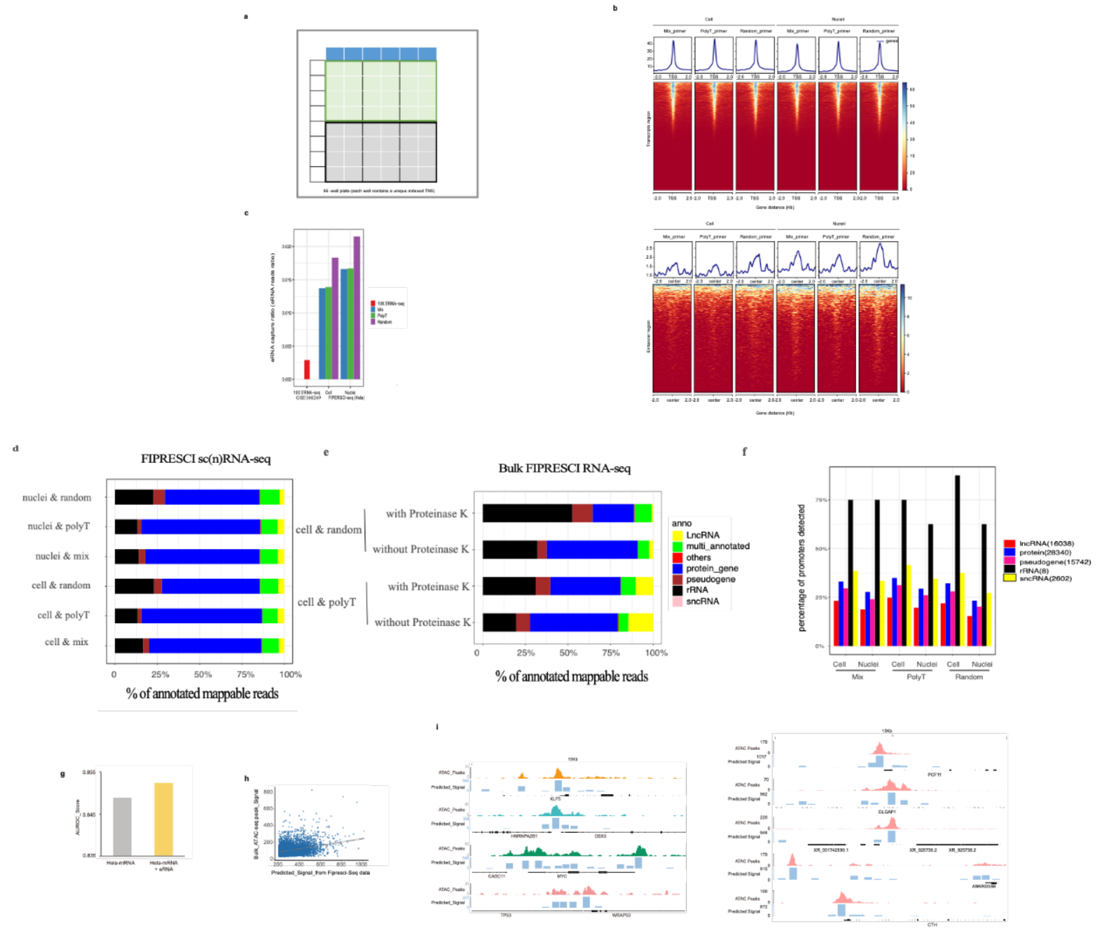

Fig. S5 | FIPRESCI enables identify TSS and enhancers.

- **a.** Design and indexed TN5 usage for different RT primer (including poly T, random, the mix of the poly T and random) experiments. Hela cell lines were used in this experiment.
- **b.** Distribution of FIPRESCI reads around annotated TSS (upper) and enhancers (lower).
- **c.** The eRNAs capture ratio. The results are calculated by #reads of 5'-end RNA-seq which overlap with distal ATAC peaks defined enhancer region/total number of reads.
- **d.** Different category of RNA detected in three reverse transcription primer conditions in FIPRESCI scRNA-seq and snRNA-seq.
- **e.** Bulk FIPRESCI RNA-seq testing with or without Proteinase K in lysis buffer.
- **f.** Percentage of promoters detected in three reverse transcription primer conditions in FIPRESCI scRNA-seq and snRNA-seq.
- **g.** The AUROC of pairing ATAC and FIPRESCI by BABEL (AUROC=0.8489) compared with ATAC and FIPRESCI combined with eRNA by BABEL (AUROC=0.8524).
- **h.** The correlation (Pearson coefficient = 0.33) of top 6,000 predict peak signal from FIPRESCI data between corresponding bulk ATAC-seq peak signal.
- **i.** Aggregate accessibility profiles for bulk ATAC-seq and predicted peak (500bp)

from FIPRESCI data around representative genes. The blue bar height represents the strength of the predicted peak signal.

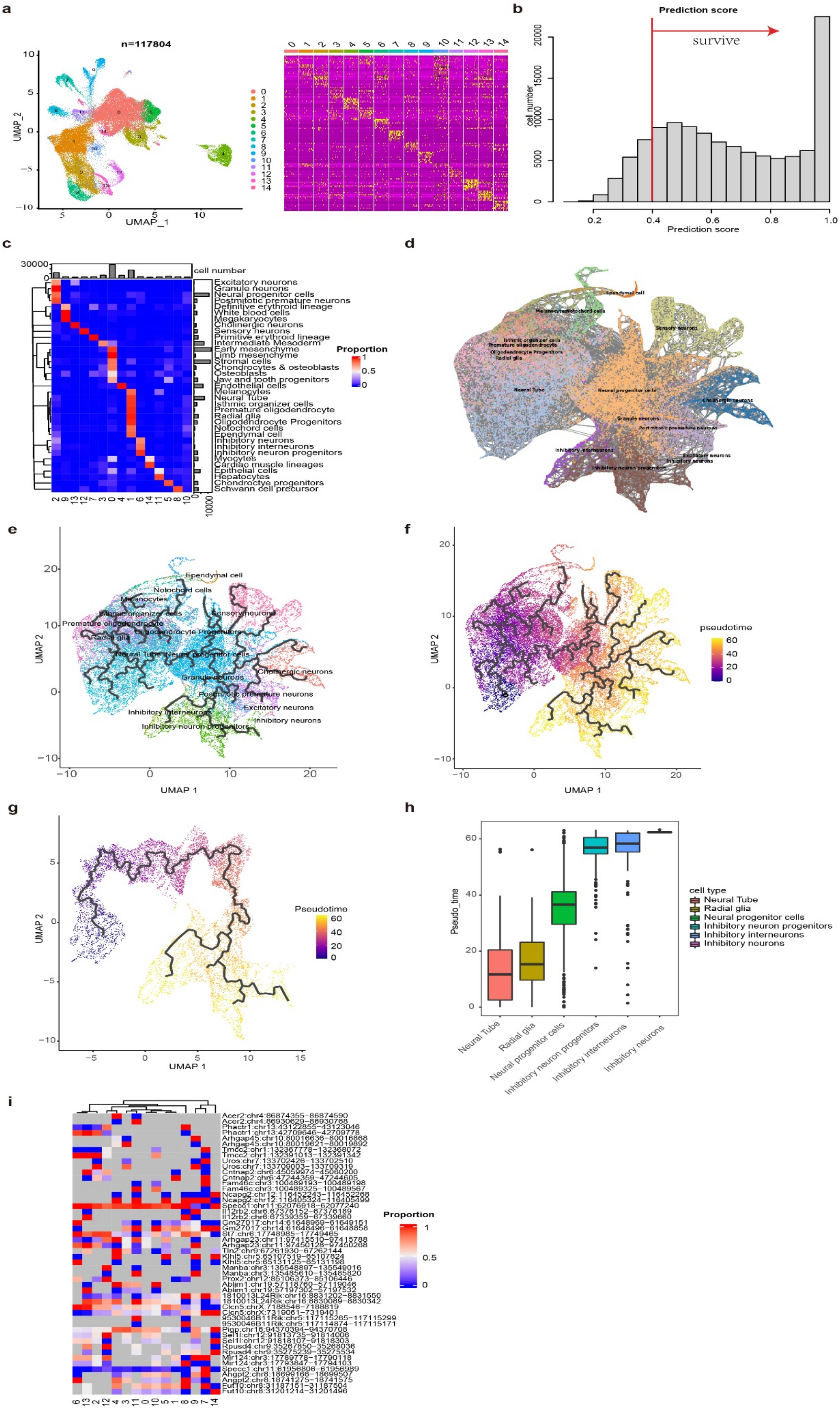
a
n=117804
UMAP_1
UMAP_2
b
Prediction score
survive
cell number
Prediction score
c
cell number
Excitatory neurons
Granule neurons
Neural progenitor cells
Postmitotic premature neurons
Definitive erythroid lineage
White blood cells
Megakaryocytes
Cholinergic neurons
Sensory neurons
Primitive erythroid lineage
Intermediate Mesoderm
Early mesenchyme
Limb mesenchyme
Stromal cells
Chondrocytes & osteoblasts
Osteoblasts
Jaw and tooth progenitors
Endothelial cells
Melanocytes
Neural Tube
Isthmic organizer cells
Premature oligodendrocyte
Radial glia
Oligodendrocyte Progenitors
Notochord cells
Ependymal cell
Inhibitory neurons
Inhibitory interneurons
Inhibitory neuron progenitors
Myocytes
Cardiac muscle lineages
Epithelial cells
Hepatocytes
Chondroctye progenitors
Schwann cell precursor
Proportion
d
e
UMAP 1
UMAP 2
f
pseudotime
g
Pseudotime
h
Pseudo_time
cell type
Neural Tube
Radial glia
Neural progenitor cells
Inhibitory neuron progenitors
Inhibitory interneurons
Inhibitory neurons
i
Proportion

Fig. S6

Fig. S6 | Details in analysis of E10.5 mouse embryo high throughput Fipresci-Seq.

a. Unsupervised clustering of 117,840 E10.5 mouse embryo cells reveals distinct clusters. UMAP embedding colored by clusters (left) and heatmap exhibiting differential expressed genes for each cluster (right).

b. Histogram of prediction score distribution in label transfer procedure. Cells with prediction greater than 0.4 survived.

c. Comparison of transferred labels (annotation) and unsupervised clusters. Colors indicated percentages of transferred annotations cells in unsupervised clusters. Bar plots showing cells numbers.

d. PAGA initiated UMAP embedding of only brain cells.

e. De novo constructing brain trajectory graph.

f. UMAP embedding of brain cells colored by pseudo time calculated by monocle3.

g. UMAP embedding of only inhibitory neuron trajectory cells colored by pseudo time inherited from f.

h. Box plot showing pseudo time distribution of inhibitory neuron trajectory cells.

i. Differential TSS usage for unsupervised clusters. Heatmap Rows represent clusters, while columns represent TSSs. Colors indicate TSS reads proportion of all TSS reads in corresponding gene within one cluster (gray color represent missing data, ordered by variance and p value<0.001).

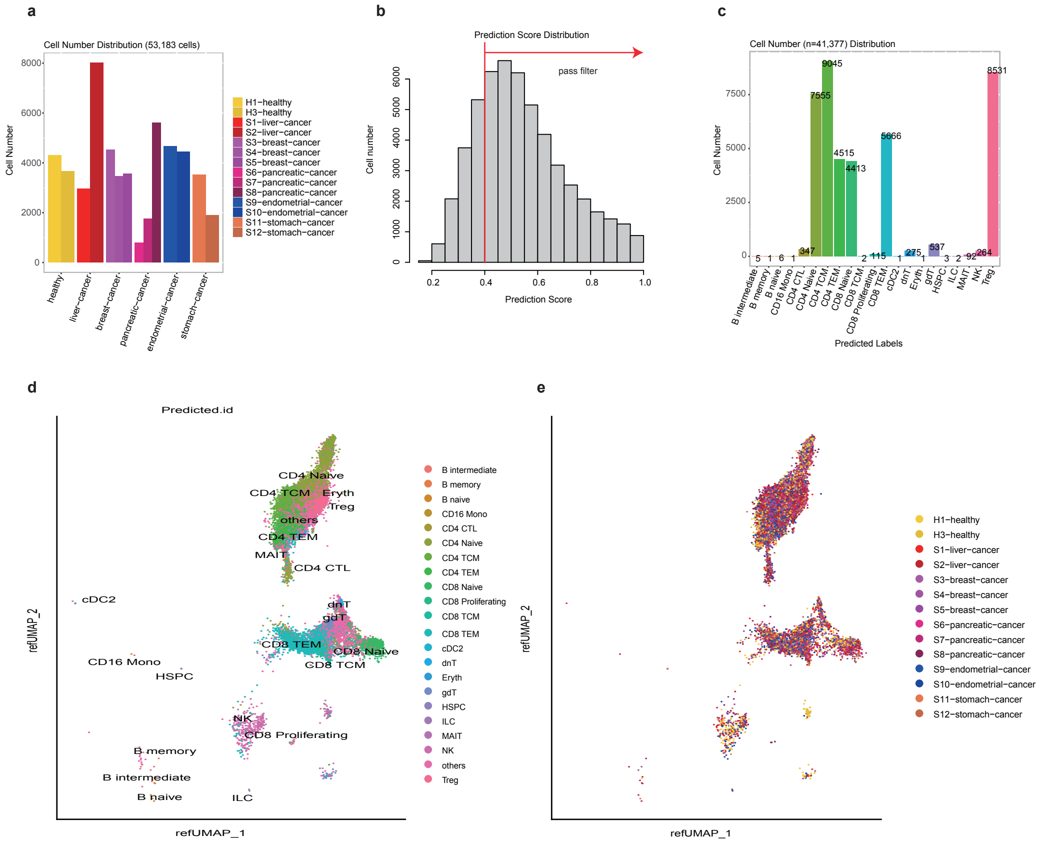

Fig. S7 | Integration analysis of FIPRESCI PBMC data and reference PBMC atlas data.

a. Bar plot showing cell numbers distribution across donors after filtering.

b. Prediction score distribution during label transfer.

c. Cell number distribution across different major immune cell types after label transfer.

d. UMAP plot showing cells colored by immune cell types in reference UMAP embedding after label transfer.

e. UMAP plot showing cells colored by donors in reference UMAP embedding after label transfer.

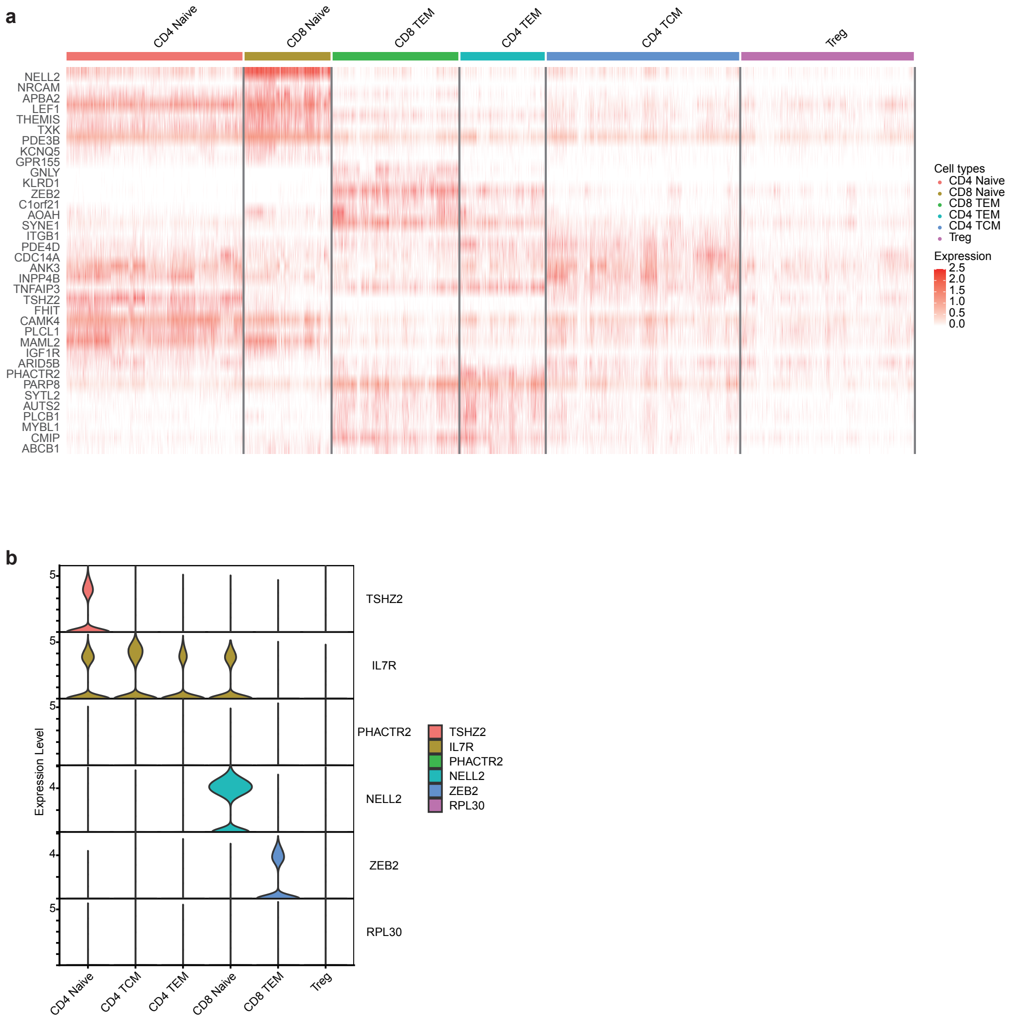

Fig. S8 | Maker genes of T cells identified in FIPRESCI.

a. Heatmap showing expression levels of selected signature genes in T cell. Each column represents a single cell, colored based on normalized expression levels.

b. Violin plots showing the marker genes expression for the major T cell types.

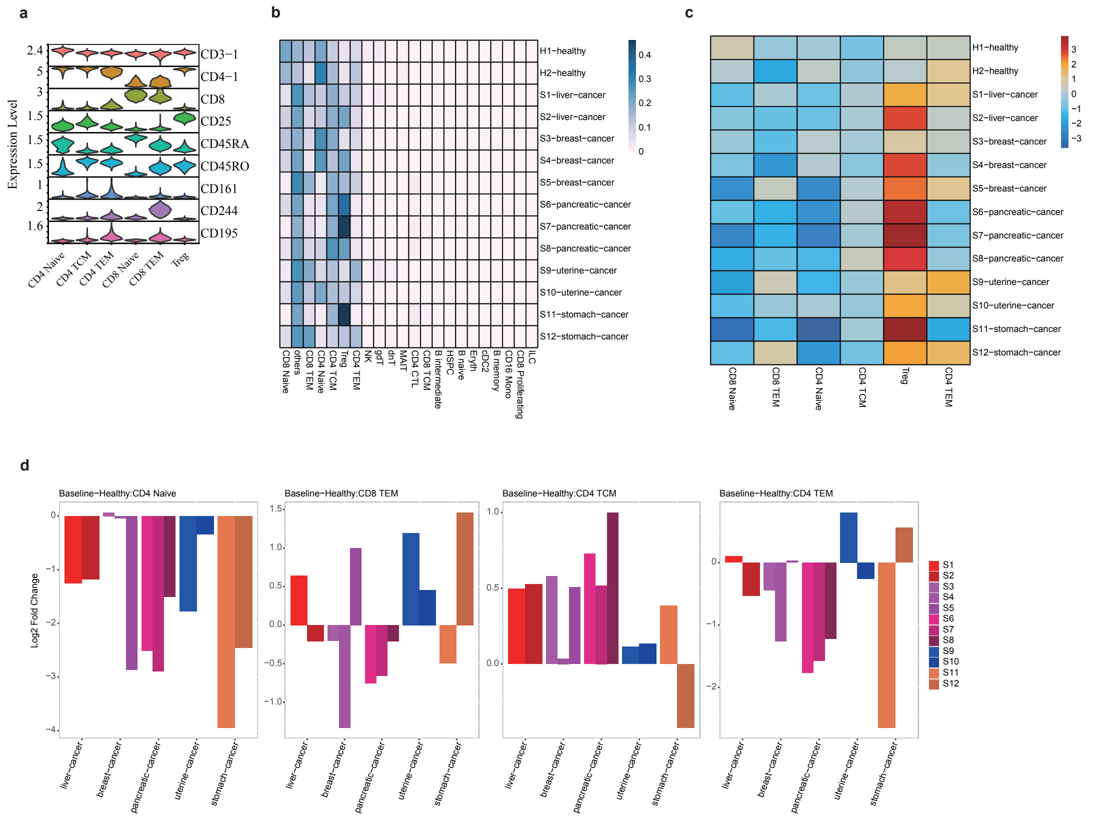

Fig. S9 | Proportion changes of T cell subtypes between cancer donors and healthy donors.

a. Violin plots showing the marker genes expression (surface proteins expression levels are imputed with PBMC CITE-seq datasets) for the major T cell subtypes.

b. Heatmap showing immune cell types proportion across different donors.

c. Heatmap showing Log2 fold changes of proportion of major T cell subtypes compared FIPRESCI with healthy donor data from reference PBMC atlas.

d. Bar plots showing Log2 fold changes of proportion of major T cell subtypes compared canner with healthy donors within FIPRESCI data.

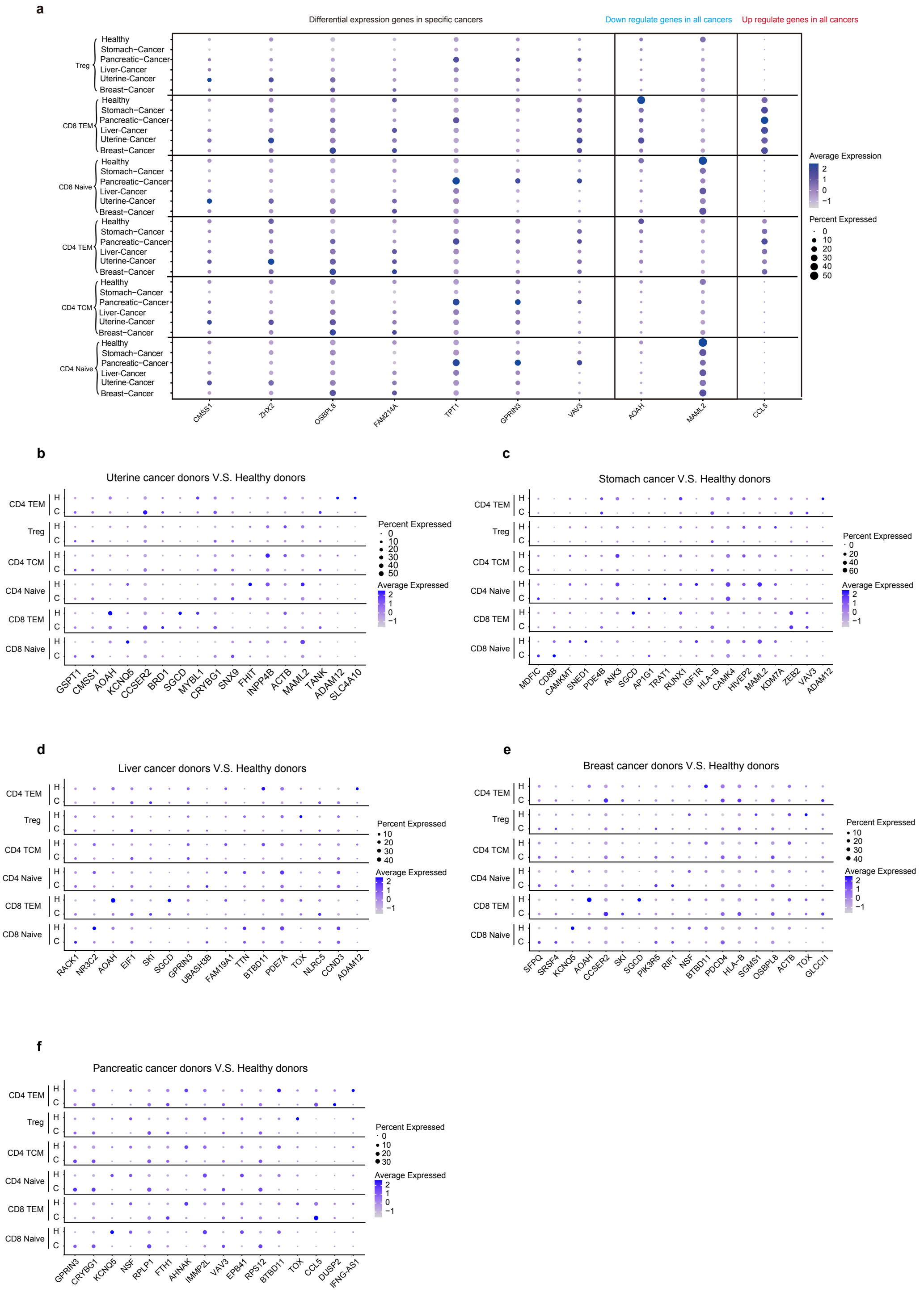

Fig. S10 | Differential expressed genes analysis compared cancer with healthy donors.

a. Bubble heatmap showing expression levels of selected signature genes. Dot size indicates the fraction of expressing cells, colored based on normalized expression levels.

b. Bubble heatmap showing differential expressed genes between uterine cancer donors and healthy donors. Dot size indicates fraction of expressing cells, colored based on normalized expression levels.

- **c.** Bubble heatmap showing differential expressed genes between stomach cancer donors and healthy donors. Dot size indicates fraction of expressing cells, colored based on normalized expression levels.
- **d.** Bubble heatmap showing differential expressed genes between liver cancer donors and healthy donors. Dot size indicates fraction of expressing cells, colored based on normalized expression levels.
- **e.** Bubble heatmap showing differential expressed genes between breast cancer donors and healthy donors. Dot size indicates fraction of expressing cells, colored based on normalized expression levels.
- **f.** Bubble heatmap showing differential expressed genes between pancreatic cancer donors and healthy donors. Dot size indicates fraction of expressing cells, colored based on normalized expression levels.

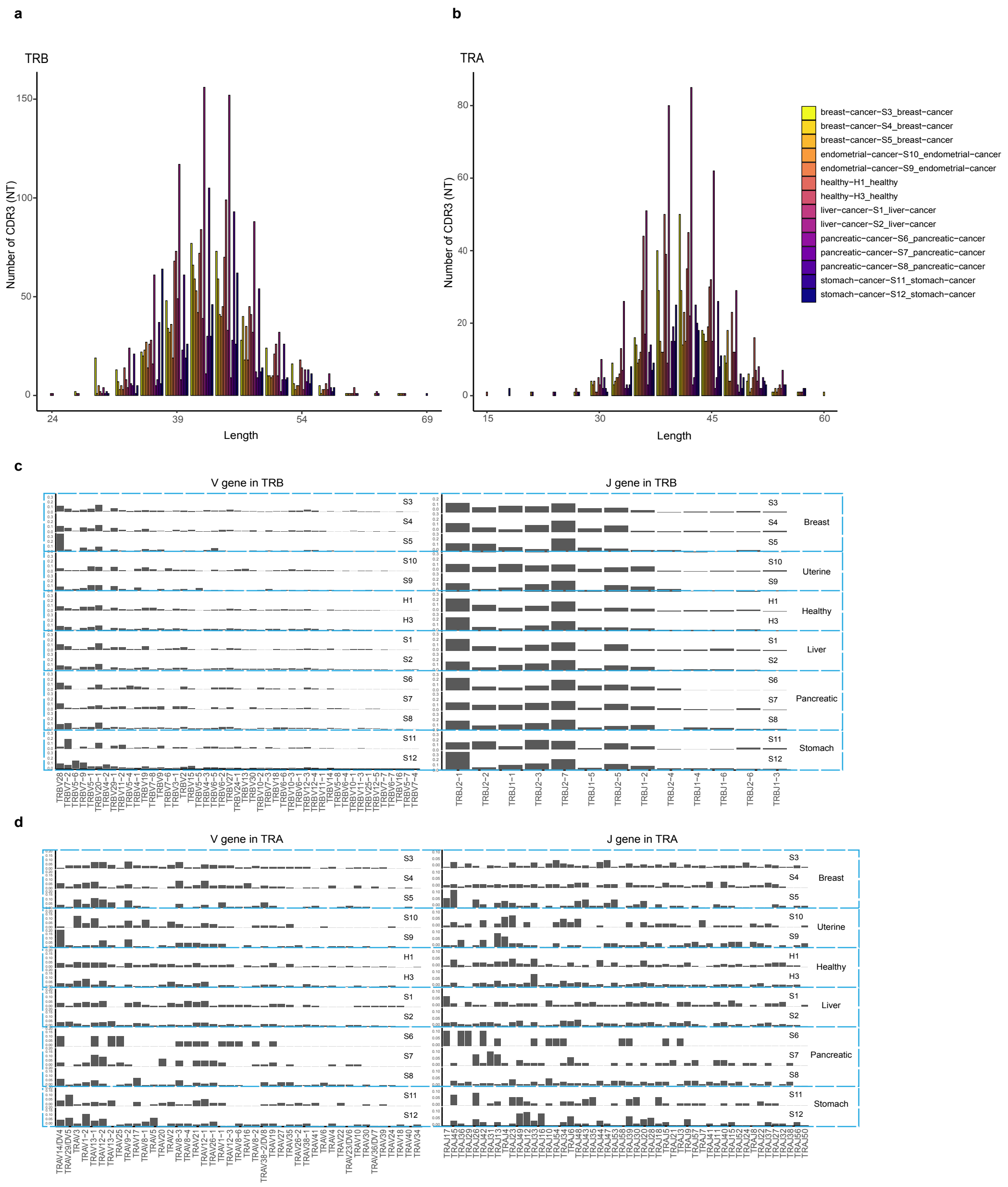

Fig. S11 | Characteristics in TRA and TRB chains

a. Length distribution of CDR3 sequences in TRA chain, colored by different donors.

b. Length distribution of CDR3 sequences in TRB chain, colored by different donors.

c. Left: V gene usages in TRB chain. V genes in x-axis are ordered by frequency variance between different donors; Right: J gene usages in TRB chain. J genes in x-axis are ordered by frequency variance between different donors.

d. Left: V gene usages in TRA chain. V genes in x-axis are ordered by frequency variance between different donors; Right: J gene usages in TRA chain. J genes in x axis are ordered by frequency variance between different donors.

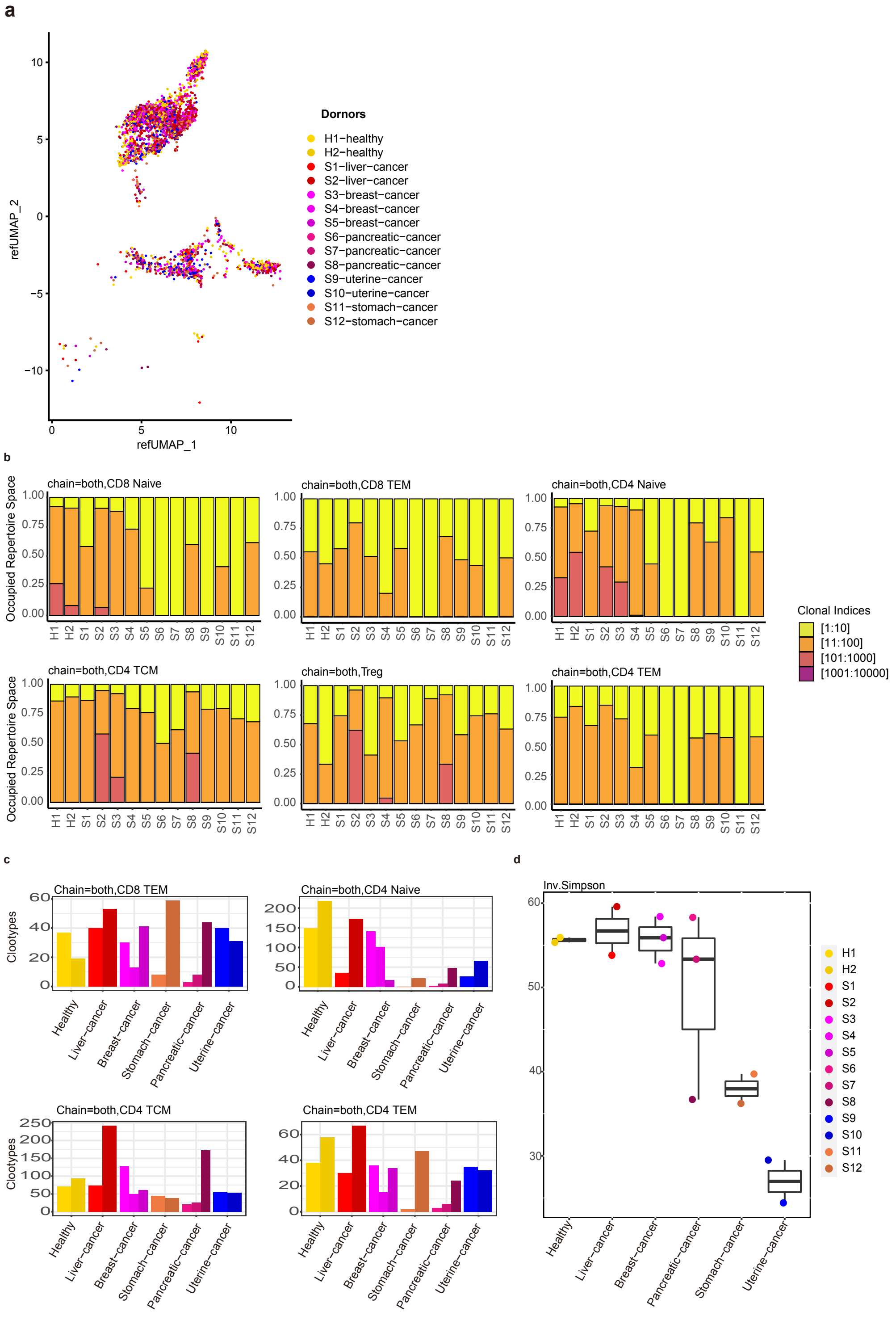

Fig. S12 | TCR Clonotype diversity analysis.

a. Cells with clonotypes detected were visualized by UMAP, colored by donors.

b. Bar plots showing dominant clonotype proportion. Clonotypes were ranked by their proportion within one T cell type. The size of bin [1:10] represented top10 clonotypes' proportion.

c. The number of unique clonotypes detected across different donors within one T cell

subtype. T cell subtype from left to right: CD4 TEM, CD4 Naïve, CD8 TEM and CD4 TCM.

d. Clonotype diversity is calculated as inverse Simpson index within one donor. Donors 'inverse Simpson indexes were grouped by their cancer types then visualized by a boxplot.

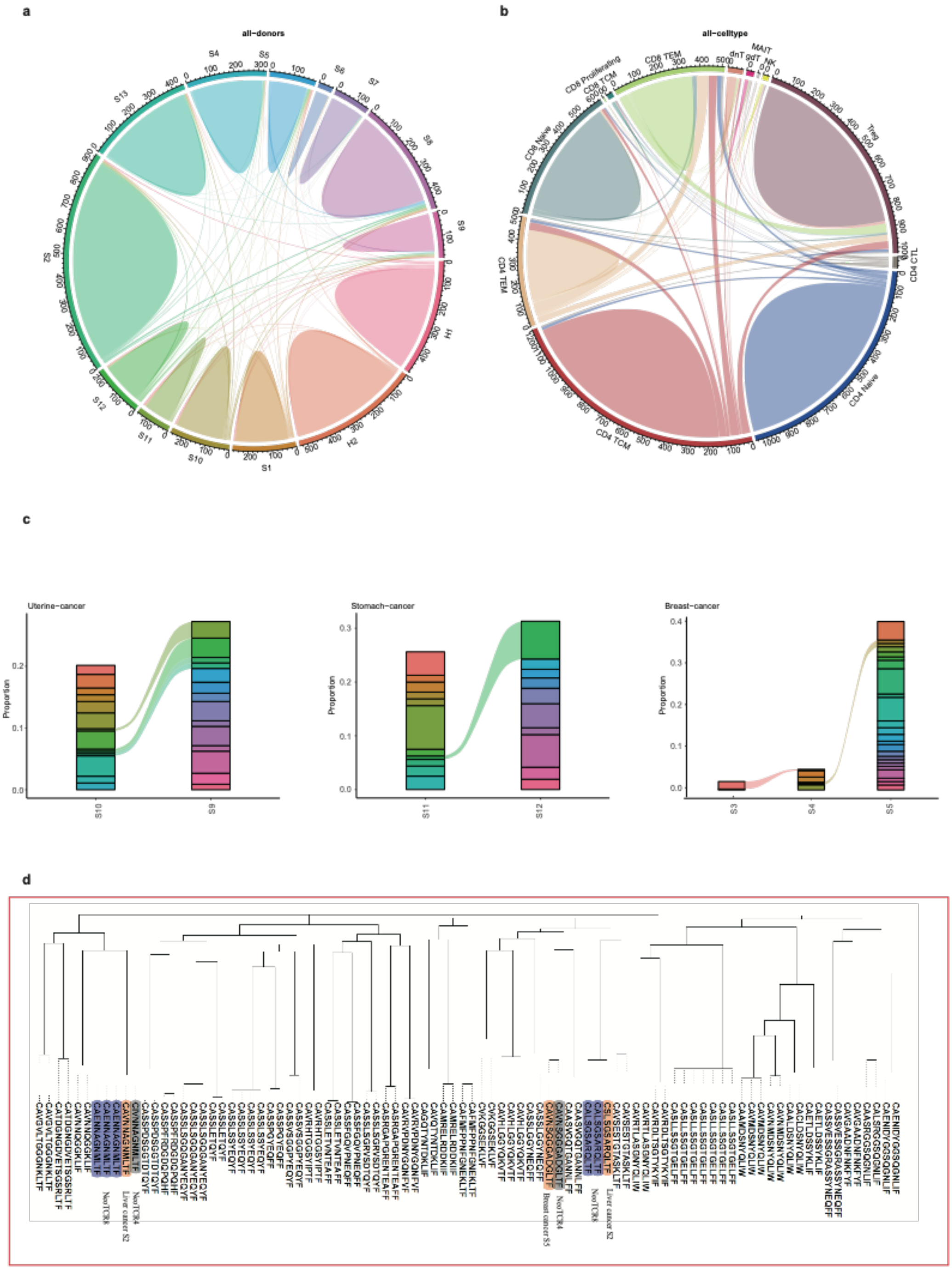

Fig. S13 | Shared clonotypes comparative analysis.

a. Shared clonotypes across all donors were visualized by Chord diagram. Links between different donors represented shared clonotypes.

b. Shared clonotypes across different immune cell types were visualized by chord diagram. Links between different immune cell types represented shared clonotypes.

c. Shared clonotypes between donors suffered from the same type of cancer. Each bin in the Sankey diagrams represented one clonotype and links represented shared clonotypes.

d. Neighbor joining tree showing TCR clusters and relationships between TCR identified from FIPRESCI and neoTCR.
