# Supplementary material for: FIPRESCI: droplet microfluidics based combinatorial indexing for massive-scale 5′-end single-cell RNA sequencing
Source: Genome Biol. 2023 Apr 6;24:70. doi: 10.1186/s13059-023-02893-1 (PMC10078054; doi:10.1186/s13059-023-02893-1)
Supplement: Supplementary file 6 — Additional file 6. Legends and web links for processed files S1-S12. [file 13059_2023_2893_MOESM6_ESM.docx]

**Legends and web links for processed files S1-S12.**

**File S1** | Human and mouse gene expression matrix from Fipresci-Seq, stored in RDS format. R package Seurat is necessary to read it.

File S1 can be downloaded from <https://figshare.com/s/3b1a127cc2dd5c2fa95a> .

**File S2** | Nuclei prepared three cell lines (Hela, HEK293T, and K562) Fipresci-Seq gene expression matrix, stored in RDS format. R package Seurat is necessary to read it.

File S2 can be downloaded from <https://figshare.com/s/e851b1bcf3f5a7102c7b> .

**File S3** | Premeabilized prepared three cell lines (Hela, HEK293T, and K562) Fipresci-Seq gene expression matrix, stored in RDS format. R package Seurat is necessary to read it.

File S3 can be downloaded from <https://figshare.com/s/f76b148e8be56d264141> .

**File S4** | Tagmentation condition test (HEK293T cell) Fipresci-Seq gene expression matrix, stored in RDS format. R package Seurat is necessary to read it.

File S4 can be downloaded from <https://figshare.com/s/d1e4f3798946f181f755> .

**File S5** | Tagmentation condition test (Hela cell) Fipresci-Seq gene expression matrix, stored in RDS format. R package Seurat is necessary to read it.

File S5 can be downloaded from <https://figshare.com/s/20beae32d95674e5d446>

**File S6** | Cell preparation and RT primer condition test Fipresci-Seq gene expression matrix, stored in RDS format. R package Seurat is necessary to read it.

File S6 can be downloaded from <https://figshare.com/s/ca6bd3c42184c6dcfd71> .

**File S7** | E10.5 mouse embryo gene expression matrix from Fipresci-Seq, stored in RDS format. R package Seurat is necessary to read it.

File S7 can be downloaded from <https://figshare.com/s/002248689f2c4e18d915> .

**File S8** | BigWig files grouped by E10.5 mouse embryo unsupervised clusters from Fipresci-Seq.

File S8 can be downloaded from <https://figshare.com/s/c4dee1de0b0afabe1d23> .

**File S9** | BigWig files grouped by Inhibitory neuron 3 stages (early, medium, and later) from Fipresci-Seq.

File S9 can be downloaded from <https://figshare.com/s/5e76c0c7eaaa9b93574e> .

**File S10** | PBMC gene expression matrix from Fipresci-Seq, stored in RDS format. R package Seurat is necessary to read it.

File S10 can be downloaded from <https://figshare.com/s/7c1235db167d83c28533> .

**File S11** | Treg sub-population gene expression matrix from Fipresci-Seq, stored in RDS format. R package Seurat is necessary to read it.

File S11 can be downloaded from <https://figshare.com/s/168e116177128043d865> .

**File S12** | PBMC TCR information from Fipresci-Seq, including cell barcodes, corresponding clonotypes, and donor information.

File S12 can be downloaded from <https://figshare.com/s/c0cd6bf44e840776ed6e> .
